# Integrated motivational interviewing and cognitive behaviour therapy for lifestyle mediators of overweight and obesity in community-dwelling adults: a systematic review and meta-analyses

**DOI:** 10.1186/s12889-018-6062-9

**Published:** 2018-10-05

**Authors:** Stephen Barrett, Stephen Begg, Paul O’Halloran, Michael Kingsley

**Affiliations:** 1 La Trobe University, La Trobe Rural Health School, PO Box 199, Bendigo, VIC 3552 Australia; 2 La Trobe University, School of Psychology and Public Health, Bundoora, VIC 3068 Australia

**Keywords:** Health promotion, Health behaviour, Obesity, Physical activity, Body composition

## Abstract

**Background:**

The aim of this study was to investigate whether integrated motivational interviewing and cognitive behaviour therapy leads to changes in lifestyle mediators of overweight and obesity in community-dwelling adults.

**Method:**

Six electronic databases were systematically searched up to 04 October, 2017. Analyses were restricted to randomised controlled trials that examined the effect of integrated motivational interviewing and cognitive behaviour therapy on lifestyle mediators of overweight and obesity (physical activity, diet, body composition) in community-dwelling adults. Meta-analyses were conducted using change scores from baseline in outcome measures specific to the lifestyle mediators of overweight and obesity to determine standardized mean differences (SMD) and 95% confidence intervals (95% CI). The Grades of Recommendation, Assessment, Development and Evaluation approach was used to evaluate the quality of the evidence.

**Results:**

Ten randomised controlled trials involving 1949 participants were included. Results revealed moderate quality evidence that integrated motivational interviewing and cognitive behaviour therapy had a significant effect in increasing physical activity levels in community-dwelling adults (SMD: 0.18, 95% CI: 0.06 to 0.31, *p* < 0.05). The combined intervention resulted in a small, non-significant effect in body composition changes (SMD: -0.12, 95% CI: -0.24 to 0.01, *p* = 0.07). Insufficient evidence existed for outcome measures relating to dietary change.

**Discussion:**

The addition of integrated motivational interviewing and cognitive behaviour therapy to usual care can lead to modest improvements in physical activity and body composition for community-dwelling adults. The available evidence demonstrates that it is feasible to integrate MI with CBT and that this combined intervention has the potential to improve health-related outcomes.

**Conclusion:**

This review details recommendations for future research including the adoption of uniform objective outcome measures and well-defined interventions with sufficient follow-up durations and assessments of treatment fidelity.

**Electronic supplementary material:**

The online version of this article (10.1186/s12889-018-6062-9) contains supplementary material, which is available to authorized users.

## Background

The epidemic of overweight and obesity continues to rise worldwide, and constitutes a serious public health concern [1]. In 2016, more than 650 million adults were classified as obese (according to the World Health Organizations [WHO] body mass index [BMI] classification of obese ≥ 30 kg/m^2^) [2]. Overweight and obesity presents a major challenge to population health due to its intricate association with a number of chronic diseases [3]. Overweight and obese individuals experience increased morbidity, functional limitations and psychosocial problems as a result of excess adiposity [4]. Due to the increasing prevalence of overweight and obesity, developing effective treatment approaches has been identified as a research and population health priority [5].

The aetiology of obesity is complex and multifaceted [6]. However, as individual and personal choices play a critical role in the manifestation of overweight and obesity, behaviour modification and lifestyle interventions are recommended as the primary steps in overweight and obesity management [7, 8]. Interventions typically target changes in lifestyle mediators of overweight and obesity namely physical activity (PA), diet, and body composition (waist circumference; mass; body mass index) [7, 9]. Psychological strategies such as increasing motivation for change, improving self-efficacy, and self-regulatory capabilities are required for addressing the lifestyle mediators of overweight and obesity and are the best predictors of beneficial physical activity and weight outcomes [8, 10–12].

Motivational interviewing (MI) is a directive, behaviour change technique that is effective in overcoming ambivalence and increasing desire for behaviour change [13]. The principles and methods of MI address issues associated with ambivalence about behaviour change, including decreased confidence and low self-efficacy [14]. MI has well established efficacy for initiating health behaviour change [8, 14, 15], but is less effective in goal-oriented action planning, which can lead to behaviour change relapse [12, 16]. Not surprisingly, therefore, it has shown to be more effective and longer lasting when combined with other active treatments, rather than delivered alone [17]. Cognitive behaviour therapy (CBT) on the other hand, posits that therapeutic strategies designed to change maladaptive cognitions can lead to improvements in behaviours [18, 19]. CBT is most commonly used to maintain behaviour change, utilising prominent strategies around relapse prevention and self-regulation [20]. In contrast to MI, CBT has shown less effectiveness in resolving ambivalence to behaviour change [21], demonstrating its greatest efficacy when working with voluntary, motivated clients [20].

Multiple studies have identified that the main factors associated with suboptimal health behaviour adoption are the lack of motivation to change and failures in strategies to maintain behaviour change [22–24]. A recent systematic review of self-regulatory mediators indicates that a lack of autonomous motivation, self-efficacy and self-regulation skills are associated with relapses in lifestyle change intervention [9]. Findings such as this, and the respective strengths and limitations of MI and CBT alone, have led to the proposal of integrating MI and CBT (MI-CBT) into a single intervention [16, 21, 25]. MI and CBT share components that are integral elements of both techniques [26]. Both approaches emphasise working in a collaborative, directive way with clients, with a clear focus on changing behaviour [13, 16, 18, 21]. Both approaches are also understood to be most effective when focused on specific behaviours [26]. Supporting client self-efficacy and behavioural self-monitoring, key principles of MI have also been utilised in most conceptualizations of CBT [27]. It has been suggested that combined behavioural interventions offer the most effective strategy for behaviour change [28].

Several systematic reviews have demonstrated only modest effectiveness for MI alone [10, 11], and CBT alone [25] in addressing lifestyle mediators of overweight and obesity, while others have shown more promising results for behaviour change in other areas using integrated MI-CBT [29, 30], though the outcomes were not related to lifestyle mediators of overweight and obesity. Together these reviews provide some preliminary evidence that integrated MI-CBT might be effective for overweight and obesity. However, no systematic review has yet been undertaken that focuses specifically on the effectiveness of integrated MI-CBT interventions for addressing the lifestyle mediators of overweight and obesity in community-dwelling populations. The primary aim of this review was to examine the effectiveness of integrated MI-CBT for lifestyle mediators of overweight and obesity in community-dwelling adults. Given the prevalence of overweight and obesity, and the recommendations for lifestyle behaviour interventions, clinicians and researchers would benefit from a systematic review that focuses on identifying the benefits associated with the use of the intervention to effect lifestyle mediators of overweight and obesity.

## Methods

This review with meta-analysis adheres to the guidelines outlined in the Preferred Reporting Items for Systematic Reviews and Meta-Analyses (PRISMA) Statement [31]. An electronic database search was conducted in Ovid Cochrane CENTRAL, Ovid MEDLINE, Ovid EMBASE, Ovid PsycINFO, CINAHL and Elsevier Scopus from inception until 04 October 2017. Search terms were grouped into three constructs: motivational interviewing, cognitive behaviour therapy, and health behaviour change. The search terms were entered as keywords or MeSH terms where possible, and initially searched with the OR operator; search constructs were combined using the AND operator. The complete search strategy for Embase PsycINFO is presented in detail (Additional file 1: Table S1). A manual search of reference lists from relevant articles was also conducted. Reference lists of selected trials were also examined to identify other relevant publications.

To be included in the current systematic review and meta-analysis, studies had to meet the following eligibility criteria: (1) an original, randomised controlled trial; (2) written in English-language; (3) adult population; (4) community-dwelling participants; (5) no active, serious mental health conditions, typically involving a diagnosis of psychosis; (6) intervention includes integrated MI-CBT; (7) intervention has at least one component that is delivered one-to-one; (8) outcome measures include a measured change in lifestyle mediators of overweight and obesity. Data were extracted using a standardised checklist. A unanimous decision was required between two reviewers to exclude a study during both abstract and full-text review. Where there was a lack of agreement between two reviewers, the disagreement was resolved by consensus via a third reviewer.

Data describing population characteristics, settings, intervention characteristics including duration and mode of delivery, measurement and verification of treatment fidelity, control group details, follow-up times, and outcomes were extracted from the included studies. Means and standard deviations of change scores for both intervention and control groups were included in one of the extracted studies [32]. Using these change data, the correlation coefficients were calculated for the intervention group (*r* = 0.50) and control group (r = 0.50), with an average r of 0.50 [33]. For all included studies, the standard deviation of change scores from baseline in outcome measures were calculated using a correlation coefficient of 0.5 [33], and entered directly into Review Manager 5.3 (The Nordic Cochrane Centre, The Cochrane Collaboration, Copenhagen, Denmark) for analysis [34]. Analyses based on changes from baseline were used because they are more efficient and powerful than comparison of final values, through the removal of between-person variability from the analysis [33]. Sub-group analysis was conducted to determine the potentially moderating effect of number of intervention sessions on the outcomes. All analyses were repeated for pre–post test correlations set at lower (0.20) and higher (0.80) values than the calculated value of 0.50 (Additional file 2: Table S2). Potential publication bias was evaluated via funnel plots (Additional file 3).

Standardized mean differences (SMD) with 95% confidence intervals (CIs) were calculated using Review Manager 5.3 as the mean difference divided by the pooled standard deviation [33]. For dichotomous variables, odds ratio (OR) with 95% CIs were calculated using Review Manager 5.3. Meta-analyses were conducted on clinically homogenous data using a random effects model, to provide an estimate of the overall effect of integrated MI-CBT on health behaviour change [33]. Cohen suggests that a standardized mean difference of 0.2 is small, 0.5 is moderate, and 0.8 or more is large [35]. In keeping with recommendations, I^2^ was used to assess statistical heterogeneity across trials [33, 35]. Heterogeneity was considered statistically significant if the *p*-value for the Chi-square test was less than 0.10 and the I^2^ statistic was 50% or more [33, 36]. In line with recommendations, if intention to treat analysis using imputed values was reported in a trial, these data were used [33]. In regards to outcome for PA, if more than one measure of PA was reported in a trial, the measure that best reflected total activity was selected and included in the analysis. Where only medians were reported, these values were treated as means and the standard deviations were derived according to the formula: standard deviation = interquartile range/1.35 [33]. Study and outcome quality was assessed according to the GRADE approach for systematic reviews [33, 37]. Quality of evidence for meta-analyses began at the high level and was downgraded to lower levels of evidence when risk of bias, inconsistency, indirectness, imprecision or publication bias were present [33, 37].

## Results

**Fig. 1:**
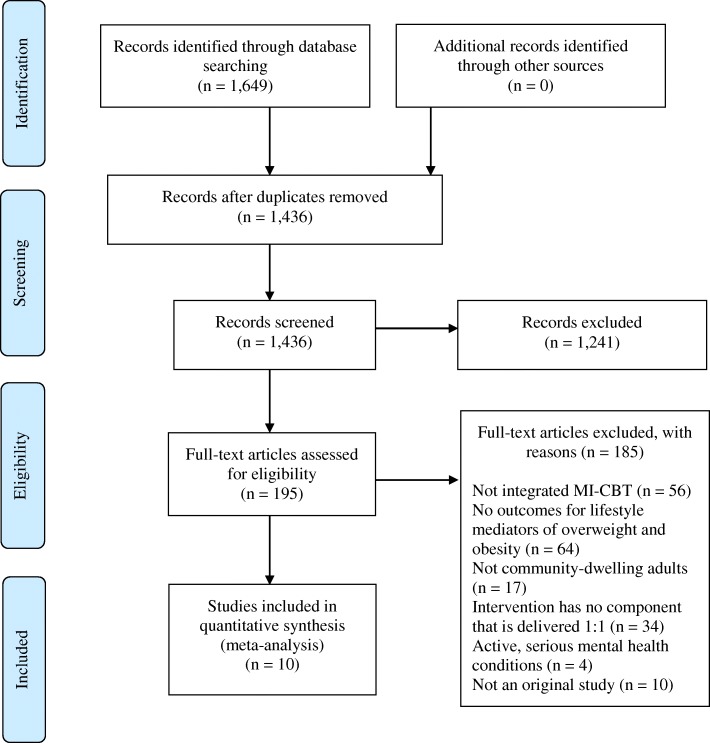
Process for identification of included trials

The literature search yielded a total of 1436 potentially relevant studies (Fig. 1). A total of 1241 studies were excluded after review of titles and abstracts, resulting in 195 studies undergoing full-text review. A total of 185 studies were excluded during full-text review leaving 10 studies remaining for data extraction. The characteristics of the included studies are listed in Table 1. All included studies were parallel randomised controlled trials that evaluated behaviour change relating to the lifestyle mediators of overweight and obesity. Of the included studies, 10 studies included an integrated MI-CBT intervention that measured PA as a behavioural outcome [32, 38–46], and 4 studies investigated integrated MI-CBT intervention for changes in body composition [40–42, 46]. The outcome measures extracted from the studies related to dietary changes were highly heterogeneous and of insufficient quality to be combined for meta-analysis.

**Table 1:** Characteristics of the included studies

Study	Country	Participants	Number of participants, *n* (% male)	Mean age at baseline (SD)	Comparator	Intervention	Outcome measures
Bennett et al., 2007 [38]	USA	Physically underactive cancer survivors	56 (10%)	57.8 ± 9.9	Advice to maintain regular physical activity	4 × 20 min telephone calls	Physical Activity: CHAMPS Physical Activity Questionnaire
Conn et al., 2003 [39]	USA	Females	190 (0%)	75.0 ± 6.7	Standard care emphasizing benefits of exercise.	1 group session plus 2 1:1 sessions^a^	Physical Activity: Baecke Physical Activity Scale
Greaves et al., 2008 [40]	UK	Community-dwelling adults	141 (42%)	51.9 (NS)	Standardised information pack promoting diet and physical activity.	11 × 30 min 1:1 sessions	Anthropometric: Measured waist circumference Physical Activity: Modifiable Activity Questionnaire
Groeneveld et al., 2011 [41]	Holland	Male construction workers	816 (100%)	46.5 ± 9.0	Standard care, consisting of brief oral or written information from the occupational physician about their CVD risk	3 × 45 min 1:1 sessions plus 4 × 15 min telephone calls	Anthropometric: Measured body massPhysical Activity: Short Questionnaire to Assess Health-Enhancing Physical Activity
Janssen et al., 2014 [42]	Holland	Former cardiac rehabilitation patients	210 (80%)	57.7 ± 9.2	1-h individual interview with a health psychologist. No motivational interviewing techniques were used	7 × 2 h group sessions plus 1 × 1:1 session^a^	Anthropometric: Measured waist circumference Physical Activity: Pedometers
Knittle et al., 2015 [43]	Holland	Patients with rheumatoid arthritis	78 (33%)	62.0 ± 11.7	A small group educational session around the importance of physical activity for people with rheumatoid arthritis	1 group session^a^ plus 3 × 60 min 1:1 sessions and 3 × 20 min telephone calls	Physical Activity: Short Questionnaire to Assess Health-Enhancing Physical Activity
Lakerveld et al., 2013 [32]	Holland	People at risk of developing cardiovascular disease and diabetes	22 (41%)	43.5 ± 5.3	Received brochures containing health guidelines regarding physical activity	6 × 30 min 1:1 plus 3 × 30 min telephone calls	Physical Activity: Short Questionnaire to Assess Health-Enhancing Physical Activity
Marques et al., 2017 [44]	Portugal	Chronic fatigue	91 (4%)	48.1 ± 10.9	Routine consultations with assistant physician and received a flyer with information about general physical activity	2 × 1:1 sessions plus 2 x telephone calls^a^	Anthropometric: Measured waist circumference Physical Activity: Short Questionnaire to Assess Health-Enhancing Physical Activity
Martens et al., 2012 [45]	USA	College students	70 (18%)	19.6 ± 2.3	Participants received informational packets about exercise	1 × 30 min 1:1 session:	Physical Activity: 7-day recall self-reported physical activity
Murphy et al., 2013 [46]	Australia	Patients with cardiovascular disease	275 (86%)	59.0 ± 9.1	Usual medical care	8 × 90 min 1:1 sessions	Physical Activity: 8-item Active Australia Survey

*CHAMPS* Community Healthy Activities Model Program for Seniors, *NS* Not stated, *UK* United Kingdom, *USA* United States of America

^a^Intervention duration not stated

Follow-up duration varied amongst the included articles; 3 studies had a 12-month follow-up [32, 41, 44], 4 studies lasted 6 months [38, 40, 42, 43], and the remaining 3 studies had a follow-up of 4 months [46], 3 months [39], and one 1 month respectively [45]. For PA outcomes, objective measures were employed in 1 study using a pedometer [41], while self-reported instruments were used in the other 9 studies [32, 38–40, 42–46]. The measures of body composition in the reviews included mass [41] and waist circumference [40, 42, 46]. Professional background of the persons delivering the intervention included PA counsellors [38], nurses [32, 41], occupational physicians [41], psychologists [42, 44], graduate students in psychology [45], health counsellors [40] and post-graduate students in sports and health science [40]. The most common methods of MI described in the studies included MI microskills (open-ended questions; affirmations; reflections; summaries) as well as feedback, affirmation and expressions of empathy. The CBT components described in the studies included problem solving, goal setting, action planning, relapse prevention, progress-related feedback and barrier identification.

**Fig. 2:**
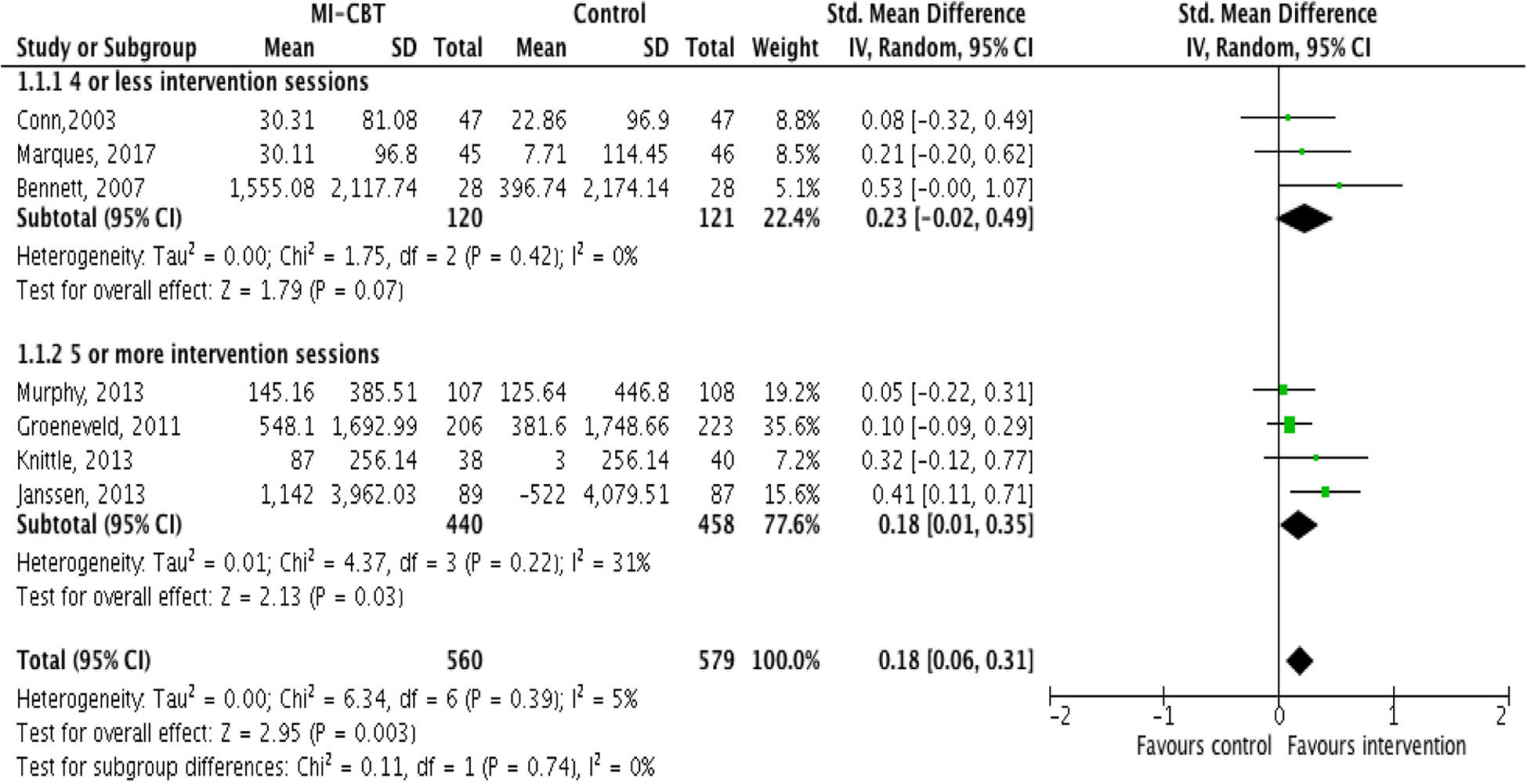
Meta-analysis investigating MI-CBT for physical activity change

**Fig. 3:**
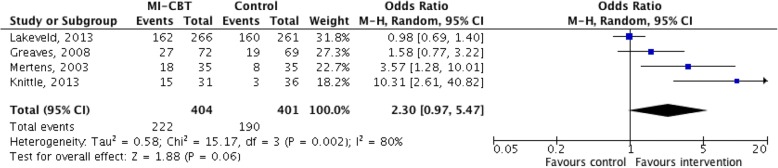
Meta-analysis investigating MI-CBT for achieving physical activity guidelines

**Fig. 4:**
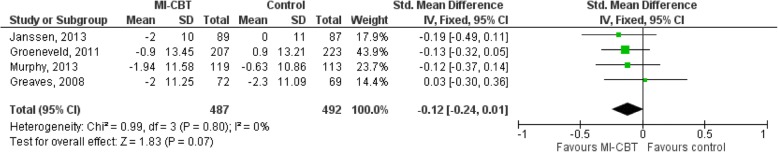
Meta-analysis investigating MI-CBT for anthropometric change

The meta-analysis for MI-CBT versus standard care for change in PA demonstrated a ‘moderate’ quality of evidence (Additional file 4: Table S3) with a significant effect in favour of the intervention (7 studies, 1139 participants, SMD, 0.18, 95% CI, 0.06 to 0.31, Fig. 2) [38, 39, 41–44, 46]. There was a ‘low’ quality of evidence, with a significant effect in favour of integrated MI-CBT when the intervention lasted for 5 sessions or more (4 studies, 898 participants, SMD, 0.18, 95% CI, 0.01 to 0.35, Fig. 2) [41–43, 46]. Interventions lasting 4 sessions or less demonstrated a ‘low’ quality of evidence with a non-significant effect in favour of integrated MI-CBT (3 studies, 241 participants, SMD, 0.23, 95% CI, -0.02 to 0.49, Fig. 2) [38, 39, 44]. The meta-analysis for integrated MI-CBT versus standard care for achieving PA guidelines demonstrated a ‘low’ quality of evidence (Additional file 4: Table S3) with a significant effect in favour of the intervention (4 studies, 805 participants, OR, 1.36, 95% CI, 1.02 to 1.81, Fig. 3) [32, 40, 43, 45]. The meta-analysis for integrated MI-CBT versus standard care for change in anthropometric measures demonstrated a ‘moderate’ quality of evidence (Additional file 4: Table S3) with a non-significant effect in favour of integrated MI-CBT (4 studies, 979 participants, SMD, -0.12, 95% CI, -0.24 to 0.01, Fig. 4) [40–42, 46].

A sensitivity analysis of the imputed correlation coefficient revealed that effect sizes remained within the 95% confidence interval. Funnel plots were consulted and indicated that potential publication bias could be ruled out.

## Discussion

This is the first systematic review and meta-analyses to analyse the effectiveness of integrated MI-CBT for lifestyle mediators of overweight and obesity. The results provide moderate quality evidence that integrated MI-CBT has a significant, beneficial effect on PA levels, and a small, beneficial effect on body composition in community-dwelling adults. This has important implications for clinicians looking to address overweight and obesity given that even small increases in PA [47] and small changes in body composition [48] can deliver beneficial health outcomes. These findings are consistent with a previous meta-analysis reporting small but clinically significant effects for integrated MI-CBT for changes in alcohol intake [30]. Stratification of the meta-analysis for MI-CBT versus standard care for change in PA by number of intervention sessions provided low quality of evidence that interventions lasting 5 sessions or more resulted in small, significant effects on PA change (Fig. 2). Interventions lasting 4 sessions or less demonstrated a ‘low’ quality of evidence with a non-significant effect in favour of integrated MI-CBT (Fig. 2). This systematic search failed to yield nutritional data of sufficient quality for inclusion in the synthesis.

The results of the meta-analysis of 7 randomised controlled trials for MI-CBT versus standard care for change in PA demonstrated moderate quality of evidence with a significant effect in favour of the intervention. Incorporating exercise as a regular lifestyle behaviour is difficult for many individuals [49]. There are multiple reasons behind this, including low motivation, poor exercise tolerance, and a lack of self-efficacy and coping skills [49]. For individuals to positively influence obesity, they must engage regularly in PA, and maintain this behaviour over a prolonged period of time [48, 49]. The integration of MI-CBT combines evidence informed strategies for motivation and maintenance of PA behaviour change. Of the 7 studies in this meta-analysis, only 4 had a change in PA as the primary outcome [38, 39, 43, 46]. Behaviour change interventions are most effective when they target single outcomes [10]. The lack of focus on PA change as the primary outcome in the studies included might account for the modest post-treatment effect size. In this meta-analysis, the downgrading of evidence to moderate was primarily due to a lack of sufficient methodological detail around the blinding of participants and personnel involved in the studies, resulting in an unclear risk of bias (Additional file 5: Table S4). Nevertheless, given the complexity and feasibility of blinding participants and personnel in studies using behaviour change techniques, the moderate level of evidence provides reasonably robust data supporting the use of integrated MI-CBT for PA change in community-dwelling adults.

Subgroup analysis for the effect of MI-CBT versus standard care for change in PA, stratified by number of intervention sessions demonstrated that interventions lasting 5 sessions or more resulted in a small but significant change in PA. Interventions lasting 4 sessions or less did not have a statistically significant effect on PA change. Treatment effect sizes for both MI alone [50] and CBT alone [51] have been shown to increase with higher numbers of intervention sessions, though the optimal treatment number remains unclear [50, 51]. Increased number of treatment sessions can strengthen skills around relapse prevention and self-management [10, 50, 51], which may contribute to the clinically significant outcomes found with 5 sessions or more [10, 50]. The broad application of behaviour change interventions in the community setting has been impeded by the lack of evidence pertaining to the optimal number of treatment sessions [52]. These meta-analyses indicate that beneficial outcomes can be derived from a small number of sessions, with increasing effect size found with 5 sessions or more.

The meta-analysis of 4 randomized controlled trials investigating MI-CBT versus standard care for achieving PA guidelines indicated that the intervention was effective at increasing PA levels in order to achieve recommended level of PA. For adults, the attainment of PA levels that approximate the recommendations for moderate activity is associated with a lower risk of mortality [53] and chronic disease [54]. In order to address overweight and obesity, the minimum of 150 min/week of moderate intensity exercise is required [55]. The primary measure of PA in the included studies was standard PA units, minutes per day or steps per day, for example. From this, the authors deduced the binary outcome of attaining or not attaining sufficient PA to meet the guidelines. No study in the meta-analysis investigating MI-CBT versus standard care for achieving PA guidelines provided any indication that participants were set a specific target of achieving the required minutes to meet the PA guidelines. This lack of homogeneity in study design and outcome measures in the included articles might reflect the high degree of heterogeneity found in the meta-analysis. While the meta-analysis indicates a positive effect of the intervention, the high heterogeneity and wide confidence intervals resulted in the downgrading of the quality of the evidence to low. In spite of these inconsistencies, the meta-analysis investigating MI-CBT versus standard care for achieving PA guidelines further supports the use of integrated MI-CBT for PA change. Future studies looking to measure attainment of PA guidelines should focus on clear outcome identification and uniform measurement.

The results of the meta-analysis of 4 randomized controlled trials for integrated MI-CBT versus standard care for change in anthropometric measures provided moderate quality of evidence that integrated MI-CBT has a small, positive effect on anthropometric measures (Fig. 4). Achieving long-term, sustainable changes in body composition is difficult [50]. At a minimum, the goal of obesity treatment is to prevent further weight gain [56], while minor changes in body composition are associated with decreased mortality in overweight individuals [57]. The beneficial effect on body composition demonstrated by the intervention in our analysis is promising. Positive change in body composition is a primary motivation for PA [58]. Positive changes in body shape have been shown to strengthen self-belief, resulting in PA maintenance [58]. All of the included studies had a 12-month follow-up, and all outcome measures were measured by research assistants. The use of objective measurement strengthens the quality of the evidence, with self-reported body weight and waist circumference measurement being prone to participant measurement error, and participant reporting bias [59]. Similar to PA, the studies included in this meta-analysis of MI-CBT versus standard care for change in anthropometric measures targeted change in multiple health behaviours, and changes in body composition outcomes was not a primary outcome in any study. When interventions target multiple health behaviours, and changes in body composition is not the primary outcome the application of behavioural change principles to body composition has been shown to decrease in priority [60]. This downgrading in perceived importance might account for the anthropometric treatment effect size found in the analysis [60].

Although the integration of MI and CBT is not a new concept [61], the confirmation and/or measurement of treatment fidelity remains difficult [61]. Fidelity scales for integrated MI-CBT have been devised and tested in the literature [61]; however none of the studies in our sample used such a fidelity measure. Therefore, the extent to which participants were actually receiving interventions is unclear, which could influence the degree of clinical homogeneity. Measurements of intervention fidelity for MI alone [62, 63] and for CBT alone [64] indicate that effect size greatly increases where treatment fidelity is measured. The lack of intervention fidelity measures in the studies included in this review may be a contributory factor to the modest effect size. Of the trials included for PA change, only two trials reported measuring fidelity of the MI component [38, 40], with the standardised mean difference for the effect of integrated MI-CBT intervention increasing from 0.18 (95% CI 0.06 to 0.30) to 0.41 (95% CI 0.07 to 0.75) when trials that did not confirm fidelity were excluded from the analysis. This effect of MI fidelity is consistent with results from previous meta-analyses which also demonstrated an increase in the standardised mean difference in trials where treatment fidelity is measured [63]. Future trials utilizing integrated MI-CBT should incorporate a measurement of fidelity into the study design.

### Strengths

This is the first systematic review and meta-analysis undertaken that provides evidence to support the use of integrated MI-CBT for changes in PA and body composition in community-dwelling adults. While the demonstrated effect was modest, the combination of MI and CBT is potentially advantageous for a number of reasons. With evidence suggesting that even small increases in PA and body composition change can deliver positive health benefits, a modest effect size, as demonstrated in this review, is likely to deliver important health outcomes [48]. The integration of MI-CBT might overcome the documented shortcomings in both interventions delivered alone, while maintaining a collaborative, directive approach [16]. While interventions incorporating behavioural or psychological components have demonstrated modest efficacy for lifestyle mediators of overweight and obesity overall, these interventions do not result in adverse effects, and generally lead to improvements in psychological well-being [65]. As single intervention studies have indicated that larger post-treatment effect sizes are produced if MI and CBT are delivered with fidelity [50, 51], it can be hypothesised that integrated MI-CBT interventions adhering to higher rates of fidelity have the potential to produce increased effect sizes [61]. Another potential advantage to using integrated MI-CBT for addressing lifestyle mediators of overweight and obesity lies in the range of health professionals that were able to deliver the intervention. This clinical diversity might be advantageous when applying the intervention across multiple sectors of the community-dwelling population, especially given the previously mentioned prevalence of overweight and obesity. For clinical interest and uptake, as indicated by the findings of these meta-analyses, higher quality randomised controlled trials with detailed interventions, extended follow-up periods, and measures of treatment fidelity are required [50, 51].

### Limitations

There are a number of limitations of our review that need to be considered. The number of included trials was restricted by the use of the rigid search criteria designed to assess the combined effects of MI-CBT on lifestyle mediators of overweight and obesity. For example, it was not possible to extract sufficient data to undertake meta-analyses for dietary changes. Excluding languages other than English might also introduce a bias and reduce the precision of estimated treatment effects. Secondly, self-reported tools were used to measure changes in PA change in 9 of the 10 included trials. This lack of an objectively measured outcomes resulted in a higher risk of bias [59]. Thirdly, this review and meta-analyses included a number of small trials, undertaken on restrictive populations, which might have influenced the observed effect sizes [33]. Finally, there may have been an impact on external validity from combining data from studies on participants with diverse health conditions. Nevertheless, for all but one of the meta-analyses, heterogeneity was low, and research continually indicates that increasing PA and positive body composition changes have favourable health effects for the majority of the population [47].

## Conclusions

Despite the small number of high quality randomised trials, this analysis indicates that integrated MI-CBT leads to modest improvements in PA and body composition changes amongst community-dwelling adults.

The emerging evidence to support the use of MI-CBT interventions for promoting the adoption and maintenance of health behaviour change has potential importance in addressing the high rates of obesity. The intervention can be delivered by a range of health professionals and can be incorporated readily into clinical practice. In order to make stronger recommendations regarding the effectiveness of this intervention for lifestyle mediators of overweight and obesity, more high quality randomised controlled trials are required. Such studies should include sufficient follow-up durations to determine the long-term effects of the interventions. Finally, trials should also include clearly defined interventions, objective outcome measures and assessments of treatment fidelity.

## Additional files

Additional file 1: **Table S1.** Search strategy and results: Ovid PsycINFO. (DOCX 44 kb)

Additional file 2: **Table S2.** Sensitivity analyses of imputed correlation coefficients for meta-analyses investigating MI-CBT for physical activity change and anthropometric change. (DOCX 12 kb)

Additional file 3: Funnel plots of meta-analyses investigating MI-CBT for physical activity change and anthropometry change. (DOCX 2897 kb)

Additional file 4: **Table S3.** Quality of evidence of Integrated Motivational Interviewing and Cognitive-Behaviour Therapy compared to standard care for physical activity change and anthropometric change. (DOCX 19 kb)

Additional file 5: **Table 4.** Risk of bias for included studies. (DOCX 13 kb)

## Abbreviations

BMI: Body Mass Index
CBT: Cognitive Behaviour Therapy
CI: Confidence Interval
MI: Motivational Interviewing
MI-CBT: Integrated MI and CBT
PA: Physical Activity
SMD: Standardized Mean Differences
