# Supplementary material for: Integrated motivational interviewing and cognitive behaviour therapy for lifestyle mediators of overweight and obesity in community-dwelling adults: a systematic review and meta-analyses
Source: BMC Public Health. 2018 Oct 5;18:1160. doi: 10.1186/s12889-018-6062-9 (PMC6173936; doi:10.1186/s12889-018-6062-9)
Supplement: Supplementary file 1 — Table S1. Search strategy and results: Ovid PsycINFO. (DOCX 44 kb) [file 12889_2018_6062_MOESM1_ESM.docx]

**Additional File 1.** Table S1: Search strategy and results: Ovid PsycINFO

|  | | | |
| --- | --- | --- | --- |
| **#** | **Searches** | **Results** | **Search Type** |
| 1 | exp Motivational Interviewing/ | 2066 | Advanced |
| 2 | motivational interviewing.mp. [mp=title, abstract, heading word, table of contents, key concepts, original title, tests & measures] | 3458 | Advanced |
| 3 | motiv* interv*.mp. [mp=title, abstract, heading word, table of contents, key concepts, original title, tests & measures] | 4074 | Advanced |
| 4 | motivat* Counsel*.mp. [mp=title, abstract, heading word, table of contents, key concepts, original title, tests & measures] | 124 | Advanced |
| 5 | health* coach*.mp. [mp=title, abstract, heading word, table of contents, key concepts, original title, tests & measures] | 169 | Advanced |
| 6 | Motivat* change.mp. [mp=title, abstract, heading word, table of contents, key concepts, original title, tests & measures] | 221 | Advanced |
| 7 | Motivat* Intervention.mp. | 431 | Advanced |
| 8 | 1 or 2 or 3 or 4 or 5 or 6 or 7 | 4526 | Advanced |
| 9 | exp Cognitive Therapy/ or exp Treatment Outcomes/ or exp Cognitive Behavior Therapy/ or exp Intervention/ | 145696 | Advanced |
| 10 | cogni* behav* ther*.mp. [mp=title, abstract, heading word, table of contents, key concepts, original title, tests & measures] | 25046 | Advanced |
| 11 | cognitive*.mp. [mp=title, abstract, heading word, table of contents, key concepts, original title, tests & measures] | 462468 | Advanced |
| 12 | behavioral.mp. [mp=title, abstract, heading word, table of contents, key concepts, original title, tests & measures] | 291117 | Advanced |
| 13 | behavioural.mp. [mp=title, abstract, heading word, table of contents, key concepts, original title, tests & measures] | 41467 | Advanced |
| 14 | cogni* behav* strat*.mp. [mp=title, abstract, heading word, table of contents, key concepts, original title, tests & measures] | 457 | Advanced |
| 15 | CBT.mp. [mp=title, abstract, heading word, table of contents, key concepts, original title, tests & measures] | 11453 | Advanced |
| 16 | CBS.mp. [mp=title, abstract, heading word, table of contents, key concepts, original title, tests & measures] | 856 | Advanced |
| 17 | maintenance.mp. [mp=title, abstract, heading word, table of contents, key concepts, original title, tests & measures] | 55967 | Advanced |
| 18 | cognitive restructuring.mp. [mp=title, abstract, heading word, table of contents, key concepts, original title, tests & measures] | 2418 | Advanced |
| 19 | mindfulness.mp. [mp=title, abstract, heading word, table of contents, key concepts, original title, tests & measures] | 10106 | Advanced |
| 20 | relaxation.mp. [mp=title, abstract, heading word, table of contents, key concepts, original title, tests & measures] | 15660 | Advanced |
| 21 | RET.mp. [mp=title, abstract, heading word, table of contents, key concepts, original title, tests & measures] | 779 | Advanced |
| 22 | 9 or 10 or 11 or 12 or 13 or 14 or 15 or 16 or 17 or 18 or 19 or 20 or 21 | 853474 | Advanced |
| 23 | 8 and 22 | 2553 | Advanced |
| 24 | limit 23 to (human and english language and embase and randomized controlled trial and journal) [Limit not valid in PsycINFO; records were retained] | 1799 | Advanced |
| 25 | health* behav* chang*.mp. [mp=title, abstract, heading word, table of contents, key concepts, original title, tests & measures] | 1477 | Advanced |
| 26 | health related behav* change.mp. [mp=title, abstract, heading word, table of contents, key concepts, original title, tests & measures] | 66 | Advanced |
| 27 | exercis*.mp. [mp=title, abstract, heading word, table of contents, key concepts, original title, tests & measures] | 65515 | Advanced |
| 28 | activity.mp. [mp=title, abstract, heading word, table of contents, key concepts, original title, tests & measures] | 285535 | Advanced |
| 29 | prevent* health.mp. [mp=title, abstract, heading word, table of contents, key concepts, original title, tests & measures] | 1942 | Advanced |
| 30 | health promotion.mp. [mp=title, abstract, heading word, table of contents, key concepts, original title, tests & measures] | 27034 | Advanced |
| 31 | diet*.mp. [mp=title, abstract, heading word, table of contents, key concepts, original title, tests & measures] | 41632 | Advanced |
| 32 | nutrition*.mp. [mp=title, abstract, heading word, table of contents, key concepts, original title, tests & measures] | 26289 | Advanced |
| 33 | smok* cessat*.mp. [mp=title, abstract, heading word, table of contents, key concepts, original title, tests & measures] | 14242 | Advanced |
| 34 | physic* activ*.mp. [mp=title, abstract, heading word, table of contents, key concepts, original title, tests & measures] | 32442 | Advanced |
| 35 | alcohol*.mp. [mp=title, abstract, heading word, table of contents, key concepts, original title, tests & measures] | 125841 | Advanced |
| 36 | 25 or 26 or 27 or 28 or 29 or 30 or 31 or 32 or 33 or 34 or 35 | 526058 | Advanced |
| 37 | 24 and 36 | 942 | Advanced |

| **Search strategy and results: Ovid MEDLINE**  **)** | | | |
| --- | --- | --- | --- |
| **#** | **Searches** | **Results** | **Search Type** |
| 1 | exp Motivational Interviewing/ | 1279 | Advanced |
| 2 | motivational interviewing.mp. [mp=title, abstract, original title, name of substance word, subject heading word, keyword heading word, protocol supplementary concept word, rare disease supplementary concept word, unique identifier, synonyms] | 3677 | Advanced |
| 3 | motiv* interv*.mp. [mp=title, abstract, original title, name of substance word, subject heading word, keyword heading word, protocol supplementary concept word, rare disease supplementary concept word, unique identifier, synonyms] | 4339 | Advanced |
| 4 | motivat* Counsel*.mp. [mp=title, abstract, original title, name of substance word, subject heading word, keyword heading word, protocol supplementary concept word, rare disease supplementary concept word, unique identifier, synonyms] | 144 | Advanced |
| 5 | health* coach*.mp. [mp=title, abstract, original title, name of substance word, subject heading word, keyword heading word, protocol supplementary concept word, rare disease supplementary concept word, unique identifier, synonyms] | 528 | Advanced |
| 6 | Motivat* change.mp. [mp=title, abstract, original title, name of substance word, subject heading word, keyword heading word, protocol supplementary concept word, rare disease supplementary concept word, unique identifier, synonyms] | 148 | Advanced |
| 7 | Motivat* Intervention.mp. | 434 | Advanced |
| 8 | 1 or 2 or 3 or 4 or 5 or 6 or 7 | 5076 | Advanced |
| 9 | exp Cognitive Therapy/ or exp Treatment Outcomes/ or exp Cognitive Behavior Therapy/ or exp Intervention/ | 24373 | Advanced |
| 10 | cogni* behav* ther*.mp. [mp=title, abstract, original title, name of substance word, subject heading word, keyword heading word, protocol supplementary concept word, rare disease supplementary concept word, unique identifier, synonyms] | 14194 | Advanced |
| 11 | cognitive*.mp. [mp=title, abstract, original title, name of substance word, subject heading word, keyword heading word, protocol supplementary concept word, rare disease supplementary concept word, unique identifier, synonyms] | 314115 | Advanced |
| 12 | behavioral.mp. [mp=title, abstract, original title, name of substance word, subject heading word, keyword heading word, protocol supplementary concept word, rare disease supplementary concept word, unique identifier, synonyms] | 275356 | Advanced |
| 13 | behavioural.mp. [mp=title, abstract, original title, name of substance word, subject heading word, keyword heading word, protocol supplementary concept word, rare disease supplementary concept word, unique identifier, synonyms] | 70662 | Advanced |
| 14 | cogni* behav* strat*.mp. [mp=title, abstract, original title, name of substance word, subject heading word, keyword heading word, protocol supplementary concept word, rare disease supplementary concept word, unique identifier, synonyms] | 243 | Advanced |
| 15 | CBT.mp. [mp=title, abstract, original title, name of substance word, subject heading word, keyword heading word, protocol supplementary concept word, rare disease supplementary concept word, unique identifier, synonyms] | 9017 | Advanced |
| 16 | CBS.mp. [mp=title, abstract, original title, name of substance word, subject heading word, keyword heading word, protocol supplementary concept word, rare disease supplementary concept word, unique identifier, synonyms] | 6689 | Advanced |
| 17 | maintenance.mp. [mp=title, abstract, original title, name of substance word, subject heading word, keyword heading word, protocol supplementary concept word, rare disease supplementary concept word, unique identifier, synonyms] | 283051 | Advanced |
| 18 | cognitive restructuring.mp. [mp=title, abstract, original title, name of substance word, subject heading word, keyword heading word, protocol supplementary concept word, rare disease supplementary concept word, unique identifier, synonyms] | 785 | Advanced |
| 19 | mindfulness.mp. [mp=title, abstract, original title, name of substance word, subject heading word, keyword heading word, protocol supplementary concept word, rare disease supplementary concept word, unique identifier, synonyms] | 5007 | Advanced |
| 20 | relaxation.mp. [mp=title, abstract, original title, name of substance word, subject heading word, keyword heading word, protocol supplementary concept word, rare disease supplementary concept word, unique identifier, synonyms] | 119892 | Advanced |
| 21 | RET.mp. [mp=title, abstract, original title, name of substance word, subject heading word, keyword heading word, protocol supplementary concept word, rare disease supplementary concept word, unique identifier, synonyms] | 7761 | Advanced |
| 22 | 9 or 10 or 11 or 12 or 13 or 14 or 15 or 16 or 17 or 18 or 19 or 20 or 21 | 1000677 | Advanced |
| 23 | 8 and 22 | 1648 | 23 |
| 24 | limit 23 to (human and english language and embase and randomized controlled trial and journal) [Limit not valid in Ovid MEDLINE(R),Ovid MEDLINE(R) Daily Update,Ovid MEDLINE(R) In-Process,Ovid MEDLINE(R) Publisher; records were retained] | 552 | Advanced |
| 25 | health* behav* chang*.mp. [mp=title, abstract, original title, name of substance word, subject heading word, keyword heading word, protocol supplementary concept word, rare disease supplementary concept word, unique identifier, synonyms] | 1737 | Advanced |
| 26 | health related behav* change.mp. [mp=title, abstract, original title, name of substance word, subject heading word, keyword heading word, protocol supplementary concept word, rare disease supplementary concept word, unique identifier, synonyms] | 92 | Advanced |
| 27 | exercis*.mp. [mp=title, abstract, original title, name of substance word, subject heading word, keyword heading word, protocol supplementary concept word, rare disease supplementary concept word, unique identifier, synonyms] | 345978 | Advanced |
| 28 | activity.mp. [mp=title, abstract, original title, name of substance word, subject heading word, keyword heading word, protocol supplementary concept word, rare disease supplementary concept word, unique identifier, synonyms] | 2638994 | Advanced |
| 29 | prevent* health.mp. [mp=title, abstract, original title, name of substance word, subject heading word, keyword heading word, protocol supplementary concept word, rare disease supplementary concept word, unique identifier, synonyms] | 17387 | Advanced |
| 30 | health promotion.mp. [mp=title, abstract, original title, name of substance word, subject heading word, keyword heading word, protocol supplementary concept word, rare disease supplementary concept word, unique identifier, synonyms] | 82508 | Advanced |
| 31 | diet*.mp. [mp=title, abstract, original title, name of substance word, subject heading word, keyword heading word, protocol supplementary concept word, rare disease supplementary concept word, unique identifier, synonyms] | 680053 | Advanced |
| 32 | nutrition*.mp. [mp=title, abstract, original title, name of substance word, subject heading word, keyword heading word, protocol supplementary concept word, rare disease supplementary concept word, unique identifier, synonyms] | 349214 | Advanced |
| 33 | smok* cessat*.mp. [mp=title, abstract, original title, name of substance word, subject heading word, keyword heading word, protocol supplementary concept word, rare disease supplementary concept word, unique identifier, synonyms] | 35760 | Advanced |
| 34 | physic* activ*.mp. [mp=title, abstract, original title, name of substance word, subject heading word, keyword heading word, protocol supplementary concept word, rare disease supplementary concept word, unique identifier, synonyms] | 98042 | Advanced |
| 35 | alcohol*.mp. [mp=title, abstract, original title, name of substance word, subject heading word, keyword heading word, protocol supplementary concept word, rare disease supplementary concept word, unique identifier, synonyms] | 391506 | Advanced |
| 36 | 25 or 26 or 27 or 28 or 29 or 30 or 31 or 32 or 33 or 34 or 35 | 4069693 | Advanced |
| 37 | 24 and 36 | 313 | Advanced |

| **Search strategy and results: Ovid Cochrane** | |
| --- | --- |
| **#** | **Searches** |
| #1 | MeSH descriptor: [Motivational Interviewing] explode all trees |
| #2 | motiv* near/1 interview* |
| #3 | motiv* near/1 counsel* |
| #4 | health coach* |
| #5 | motiv* change |
| #6 | motivat* intervention* |
| #7 | #1 or #2 or #3 or #4 or #5 or #6 |
| #8 | MeSH descriptor: [Cognitive Therapy] explode all trees |
| #9 | MeSH descriptor: [Cognitive Therapy] explode all trees |
| #10 | cognit* near/1 behav* near/1 therap* |
| #11 | cognit* near/1 behav* near/1 treat* |
| #12 | cognitive* |
| #13 | behavioural |
| #14 | behavioral |
| #15 | CBT |
| #16 | cognitive behav* strat* |
| #17 | CBS |
| #18 | Maintenance |
| #19 | Cognitive restructuring |
| #20 | Mindfulness |
| #21 | Relaxation |
| #22 | RET |
| #23 | #10 or #11 or #12 or #13 or #14 or #15 or #16 or #17 or #18 or #19 or #20 or #21 or #22 |
| #24 | health behav* change |
| #25 | health related behav* change |
| #26 | prevent* health |
| #27 | health promotion |
| #28 | exercis* |
| #29 | physical activity |
| #30 | diet* |
| #31 | nutrition* |
| #32 | physic* activ* |
| #33 | smok* cess* |
| #34 | alcohol* |
| #35 | #24 or #25 or #26 or #27 or #28 or #29 or #30 or #31 or #32 or #33 or #34 |
| #36 | #7 and #23 and #35 |

| **Search strategy and results: Ovid EMBASE** | | | |
| --- | --- | --- | --- |
| **#** | **Searches** | **Results** | **Search Type** |
| 1 | exp Motivational Interviewing/ | 3205 | Advanced |
| 2 | motivational interviewing.mp. [mp=title, abstract, heading word, drug trade name, original title, device manufacturer, drug manufacturer, device trade name, keyword, floating subheading word] | 5417 | Advanced |
| 3 | motiv* interv*.mp. [mp=title, abstract, heading word, drug trade name, original title, device manufacturer, drug manufacturer, device trade name, keyword, floating subheading word] | 6281 | Advanced |
| 4 | motivat* Counsel*.mp. [mp=title, abstract, heading word, drug trade name, original title, device manufacturer, drug manufacturer, device trade name, keyword, floating subheading word] | 204 | Advanced |
| 5 | health* coach*.mp. [mp=title, abstract, heading word, drug trade name, original title, device manufacturer, drug manufacturer, device trade name, keyword, floating subheading word] | 727 | Advanced |
| 6 | Motivat* change.mp. [mp=title, abstract, heading word, drug trade name, original title, device manufacturer, drug manufacturer, device trade name, keyword, floating subheading word] | 167 | Advanced |
| 7 | Motivat* Intervention.mp. | 570 | Advanced |
| 8 | 1 or 2 or 3 or 4 or 5 or 6 or 7 | 7247 | Advanced |
| 9 | exp Cognitive Therapy/ or exp Treatment Outcomes/ or exp Cognitive Behavior Therapy/ or exp Intervention/ | 47212 | Advanced |
| 10 | cogni* behav* ther*.mp. [mp=title, abstract, heading word, drug trade name, original title, device manufacturer, drug manufacturer, device trade name, keyword, floating subheading word] | 21425 | Advanced |
| 11 | cognitive*.mp. [mp=title, abstract, heading word, drug trade name, original title, device manufacturer, drug manufacturer, device trade name, keyword, floating subheading word] | 462022 | Advanced |
| 12 | behavioral.mp. [mp=title, abstract, heading word, drug trade name, original title, device manufacturer, drug manufacturer, device trade name, keyword, floating subheading word] | 317493 | Advanced |
| 13 | behavioural.mp. [mp=title, abstract, heading word, drug trade name, original title, device manufacturer, drug manufacturer, device trade name, keyword, floating subheading word] | 90064 | Advanced |
| 14 | cogni* behav* strat*.mp. [mp=title, abstract, heading word, drug trade name, original title, device manufacturer, drug manufacturer, device trade name, keyword, floating subheading word] | 308 | Advanced |
| 15 | CBT.mp. [mp=title, abstract, heading word, drug trade name, original title, device manufacturer, drug manufacturer, device trade name, keyword, floating subheading word] | 12713 | Advanced |
| 16 | CBS.mp. [mp=title, abstract, heading word, drug trade name, original title, device manufacturer, drug manufacturer, device trade name, keyword, floating subheading word] | 7988 | Advanced |
| 17 | maintenance.mp. [mp=title, abstract, heading word, drug trade name, original title, device manufacturer, drug manufacturer, device trade name, keyword, floating subheading word] | 354884 | Advanced |
| 18 | cognitive restructuring.mp. [mp=title, abstract, heading word, drug trade name, original title, device manufacturer, drug manufacturer, device trade name, keyword, floating subheading word] | 1201 | Advanced |
| 19 | mindfulness.mp. [mp=title, abstract, heading word, drug trade name, original title, device manufacturer, drug manufacturer, device trade name, keyword, floating subheading word] | 6979 | Advanced |
| 20 | relaxation.mp. [mp=title, abstract, heading word, drug trade name, original title, device manufacturer, drug manufacturer, device trade name, keyword, floating subheading word] | 137453 | Advanced |
| 21 | RET.mp. [mp=title, abstract, heading word, drug trade name, original title, device manufacturer, drug manufacturer, device trade name, keyword, floating subheading word] | 11543 | Advanced |
| 22 | 9 or 10 or 11 or 12 or 13 or 14 or 15 or 16 or 17 or 18 or 19 or 20 or 21 | 1272417 | Advanced |
| 23 | 8 and 22 | 2529 | Advanced |
| 24 | limit 23 to (human and english language and embase and randomized controlled trial and journal) | 351 | Advanced |
| 25 | health* behav* chang*.mp. | 1810 | Advanced |
| 26 | health related behav* change.mp. | 98 | Advanced |
| 27 | exercis*.mp. | 470153 | Advanced |
| 28 | activity.mp. | 3762872 | Advanced |
| 29 | prevent* health.mp. | 31398 | Advanced |
| 30 | health promotion.mp. | 95711 | Advanced |
| 31 | diet*.mp. | 926400 | Advanced |
| 32 | nutrition*.mp. | 458818 | Advanced |
| 33 | smok* cessat*.mp. | 55908 | Advanced |
| 34 | physic* activ*.mp. | 169789 | Advanced |
| 35 | alcohol*.mp. | 614189 | Advanced |
| 36 | 25 or 26 or 27 or 28 or 29 or 30 or 31 or 32 or 33 or 34 or 35 | 5691257 | Advanced |
| 37 | 24 and 36 | 215 | Advanced |

| **Search strategy and results: Ovid CINAHL** | | | |
| --- | --- | --- | --- |
| **#** | **Searches** | **Results** | **Search Type** |
| S1 | MJ motivational interviewing | Search modes - Boolean/Phrase | Interface - EBSCOhost Research Databases  Search Screen - Advanced Search  Database - CINAHL |
| S2 | motiv* interview* | Search modes - Boolean/Phrase | Interface - EBSCOhost Research Databases  Search Screen - Advanced Search  Database - CINAHL |
| S3 | motivat* Counsel* | Search modes - Boolean/Phrase | Interface - EBSCOhost Research Databases  Search Screen - Advanced Search  Database - CINAHL |
| S4 | health coach* | Search modes - Boolean/Phrase | Interface - EBSCOhost Research Databases  Search Screen - Advanced Search  Database - CINAHL |
| S5 | Motivat* change | Search modes - Boolean/Phrase | Interface - EBSCOhost Research Databases  Search Screen - Advanced Search  Database - CINAHL |
| S6 | Motivat* Intervention | Search modes - Boolean/Phrase | Interface - EBSCOhost Research Databases  Search Screen - Advanced Search  Database - CINAHL |
| S7 | S1 OR S2 OR S3 OR S4 OR S5 OR S6 | Search modes - Boolean/Phrase | Interface - EBSCOhost Research Databases  Search Screen - Advanced Search  Database - CINAHL |
| S8 | cognitive behavioral therapy | Search modes - Boolean/Phrase | Interface - EBSCOhost Research Databases  Search Screen - Advanced Search  Database - CINAHL |
| S9 | cogni* behav* ther* | Search modes - Boolean/Phrase | Interface - EBSCOhost Research Databases  Search Screen - Advanced Search  Database - CINAHL |
| S10 | cognitive* | Search modes - Boolean/Phrase | Interface - EBSCOhost Research Databases  Search Screen - Advanced Search  Database - CINAHL |
| S11 | behavioral | Search modes - Boolean/Phrase | Interface - EBSCOhost Research Databases  Search Screen - Advanced Search  Database - CINAHL |
| S12 | behavioural | Search modes - Boolean/Phrase | Interface - EBSCOhost Research Databases  Search Screen - Advanced Search  Database - CINAHL |
| S13 | cogni* behav* strat* | Search modes - Boolean/Phrase | Interface - EBSCOhost Research Databases  Search Screen - Advanced Search  Database - CINAHL |
| S14 | cbt | Search modes - Boolean/Phrase | Interface - EBSCOhost Research Databases  Search Screen - Advanced Search  Database - CINAHL |
| S15 | CBS | Search modes - Boolean/Phrase | Interface - EBSCOhost Research Databases  Search Screen - Advanced Search  Database - CINAHL |
| S16 | maintenance | Search modes - Boolean/Phrase | Interface - EBSCOhost Research Databases  Search Screen - Advanced Search  Database - CINAHL |
| S17 | cognitive restructuring | Search modes - Boolean/Phrase | Interface - EBSCOhost Research Databases  Search Screen - Advanced Search  Database - CINAHL |
| S18 | mindfulness | Search modes - Boolean/Phrase | Interface - EBSCOhost Research Databases  Search Screen - Advanced Search  Database - CINAHL |
| S19 | relaxation | Search modes - Boolean/Phrase | Interface - EBSCOhost Research Databases  Search Screen - Advanced Search  Database - CINAHL |
| S20 | RET | Search modes - Boolean/Phrase | Interface - EBSCOhost Research Databases  Search Screen - Advanced Search  Database - CINAHL |
| S21 | S8 OR S9 OR S10 OR S11 OR S12 OR S13 OR S14 OR S15 OR S16 OR S17 OR S18 OR S19 OR S20 | Search modes - Boolean/Phrase | Interface - EBSCOhost Research Databases  Search Screen - Advanced Search  Database - CINAHL |
| S22 | health behavior change | Search modes - Boolean/Phrase | Interface - EBSCOhost Research Databases  Search Screen - Advanced Search  Database - CINAHL |
| S23 | health related behaviour change | Search modes - Boolean/Phrase | Interface - EBSCOhost Research Databases  Search Screen - Advanced Search  Database - CINAHL |
| S24 | health promotion | Search modes - Boolean/Phrase | Interface - EBSCOhost Research Databases  Search Screen - Advanced Search  Database - CINAHL |
| S25 | health promotion | Search modes - Boolean/Phrase | Interface - EBSCOhost Research Databases  Search Screen - Advanced Search  Database - CINAHL |
| S26 | prevent* health | Search modes - Boolean/Phrase | Interface - EBSCOhost Research Databases  Search Screen - Advanced Search  Database - CINAHL |
| S27 | physical* | Search modes - Boolean/Phrase | Interface - EBSCOhost Research Databases  Search Screen - Advanced Search  Database - CINAHL |
| S28 | exercis* | Search modes - Boolean/Phrase | Interface - EBSCOhost Research Databases  Search Screen - Advanced Search  Database - CINAHL |
| S29 | inactiv* | Search modes - Boolean/Phrase | Interface - EBSCOhost Research Databases  Search Screen - Advanced Search  Database - CINAHL |
| S30 | diet* | Search modes - Boolean/Phrase | Interface - EBSCOhost Research Databases  Search Screen - Advanced Search  Database - CINAHL |
| S31 | weight* | Search modes - Boolean/Phrase | Interface - EBSCOhost Research Databases  Search Screen - Advanced Search  Database - CINAHL |
| S32 | nutrition* | Search modes - Boolean/Phrase | Interface - EBSCOhost Research Databases  Search Screen - Advanced Search  Database - CINAHL |
| S33 | smoking cessation | Search modes - Boolean/Phrase | Interface - EBSCOhost Research Databases  Search Screen - Advanced Search  Database - CINAHL |
| S34 | alcohol* | Search modes - Boolean/Phrase | Interface - EBSCOhost Research Databases  Search Screen - Advanced Search  Database - CINAHL |
| S35 | S22 OR S23 OR S24 OR S25 OR S26 OR S27 OR S28 OR S29 OR S30 OR S31 OR S32 OR S33 OR S34 | Search modes - Boolean/Phrase | Interface - EBSCOhost Research Databases  Search Screen - Advanced Search  Database - CINAHL |
| S36 | S7 AND S21 AND S35 | Search modes - Boolean/Phrase | Interface - EBSCOhost Research Databases  Search Screen - Advanced Search  Database - CINAHL |
| S37 | S7 AND S21 AND S35 | Limiters - Peer Reviewed; English Language; Research Article; Human; Age Groups: All Adult  Search modes - Boolean/Phrase | Interface - EBSCOhost Research Databases  Search Screen - Advanced Search  Database - CINAHL |
