# Supplementary material for: Integrated motivational interviewing and cognitive behaviour therapy for lifestyle mediators of overweight and obesity in community-dwelling adults: a systematic review and meta-analyses
Source: BMC Public Health. 2018 Oct 5;18:1160. doi: 10.1186/s12889-018-6062-9 (PMC6173936; doi:10.1186/s12889-018-6062-9)
Supplement: Supplementary file 2 — Table S2. Sensitivity analyses of imputed correlation coefficients for meta-analyses investigating MI-CBT for physical activity change and anthropometric change. (DOCX 12 kb) [file 12889_2018_6062_MOESM2_ESM.docx]

**Additional file 2.** Table S2: Sensitivity analyses of imputed correlation coefficients for meta-analyses investigating MI-CBT for physical activity change and anthropometric change

|  | **Std. Mean Difference, Fixed (95% CI)** | | |
| --- | --- | --- | --- |
| **Outcome Measure** | **Imputed Correlation Coefficient = 0.5** | **Imputed Correlation Coefficient = 0.2** | **Imputed Correlation Coefficient = 0.8** |
| Physical activity change | 0.18 (0.06, 0.31) | 0.15 (0.03, 0.26) | 0.28 (0.16, 0.39) |
| Anthropometric change | -0.12 (-0.24,0.01) | -0.09 (-0.22, 0.03) | -0.18 (-0.31, -0.06) |

Std. Mean Difference: standardized mean difference; Fixed: fixed-effects model; (95% CI): 95% confidence interval.
