# Supplementary material for: Integrated motivational interviewing and cognitive behaviour therapy for lifestyle mediators of overweight and obesity in community-dwelling adults: a systematic review and meta-analyses
Source: BMC Public Health. 2018 Oct 5;18:1160. doi: 10.1186/s12889-018-6062-9 (PMC6173936; doi:10.1186/s12889-018-6062-9)
Supplement: Supplementary file 3 — Funnel plots of meta-analyses investigating MI-CBT for physical activity change and anthropometry change. (DOCX 2897 kb) [file 12889_2018_6062_MOESM3_ESM.docx]

**Additional file 3:** Funnel plots for meta-analyses investigating MI-CBT for physical activity change and anthropometry change.


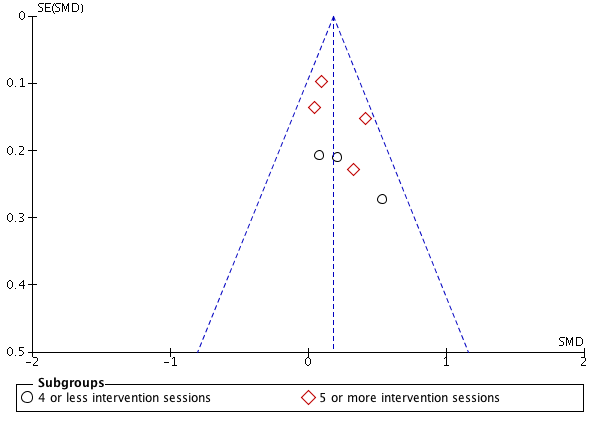


Figure a. Funnel plot for meta-analysis investigating MI-CBT for physical activity change.


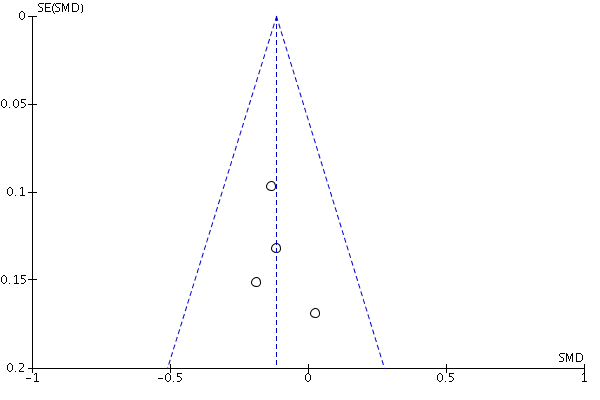


Figure b. Funnel plot for meta-analysis investigating MI-CBT for anthropometric change.


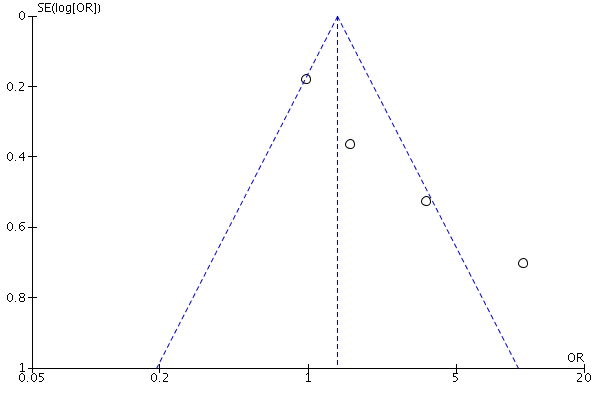


Figure c. Funnel plot for meta-analysis investigating MI-CBT for achieving physical activity guidelines.
