# Supplementary material for: Integrated motivational interviewing and cognitive behaviour therapy for lifestyle mediators of overweight and obesity in community-dwelling adults: a systematic review and meta-analyses
Source: BMC Public Health. 2018 Oct 5;18:1160. doi: 10.1186/s12889-018-6062-9 (PMC6173936; doi:10.1186/s12889-018-6062-9)
Supplement: Supplementary file 4 — Table S3. Quality of evidence of Integrated Motivational Interviewing and Cognitive-Behaviour Therapy compared to standard care for physical activity change and anthropometric change. (DOCX 19 kb) [file 12889_2018_6062_MOESM4_ESM.docx]

**Additional file 4.** Table S3: Quality of evidence

a: Integrated Motivational Interviewing and Cognitive-Behaviour Therapy compared to standard care for physical activity change.

| **Quality assessment** | | | | | | | **№ of patients** | | **Effect** | | **Quality** | **Importance** |
| --- | --- | --- | --- | --- | --- | --- | --- | --- | --- | --- | --- | --- |
| **№ of studies** | **Study design** | **Risk of bias** | **Inconsistency** | **Indirectness** | **Imprecision** | **Other considerations** | **Motivational Interviewing and Cognitive-Behaviour Therapy** | **standard care** | **Relative (95% CI)** | **Absolute (95% CI)** |  |  |
| Physical Activity Change | | | | | | | | | | | | |
| 7 | randomised trials | not serious | not serious | not serious | serious ^a^ | none | 560 | 579 | - | SMD **0.18 higher** (0.06 higher to 0.31 higher) | ⨁⨁⨁◯ MODERATE |  |
| Meeting physical activity guidelines | | | | | | | | | | | | |
| 4 | randomised trials | not serious | serious ^b^ | not serious | serious ^a^ | none | 222/404 (55.0%) | 190/401 (47.4%) | **OR 2.30** (0.97 to 5.57) | **77 more per 1,000** (from 5 more to 146 more) | ⨁⨁◯◯ LOW |  |
| Physical Activity Change Short term intervention | | | | | | | | | | | | |
| 3 | randomised trials | not serious | not serious | not serious | serious ^a^ | none | 120 | 121 | - | SMD **0.23 higher** (0.02 lower to 0.49 higher) | ⨁⨁⨁◯ MODERATE |  |
| Physical Activity Change Long term intervention | | | | | | | | | | | | |
| 4 | randomised trials | not serious | not serious | not serious | serious ^b^ | none | 440 | 458 | - | SMD **0.18 higher** (0.01 higher to 0.35 higher) | ⨁⨁⨁◯ MODERATE |  |

CI: Confidence interval; SMD: Standardised mean difference; OR: Odds ratio

a. Inadequate sample size; b. Significant Heterogeneity

b: Integrated Motivational Interviewing and Cognitive-Behaviour Therapy compared to standard care for anthropometric change

| **Quality assessment** | | | | | | | **№ of patients** | | **Effect** | | **Quality** | **Importance** |
| --- | --- | --- | --- | --- | --- | --- | --- | --- | --- | --- | --- | --- |
| **№ of studies** | **Study design** | **Risk of bias** | **Inconsistency** | **Indirectness** | **Imprecision** | **Other considerations** | **Motivational Interviewing and Cognitive-Behaviour Therapy** | **standard care** | **Relative (95% CI)** | **Absolute (95% CI)** |  |  |
| Integrated MI and CBT for body change | | | | | | | | | | | | |
| 4 | randomised trials | not serious | not serious | serious ^a^ | not serious | none | 487 | 492 | - | SMD **0.12 lower** (0.24 lower to 0.01 higher) | ⨁⨁⨁◯ MODERATE |  |

CI: Confidence interval; SMD: Standardised mean difference

a. wide confidence intervals
