# Supplementary material for: Integrated motivational interviewing and cognitive behaviour therapy for lifestyle mediators of overweight and obesity in community-dwelling adults: a systematic review and meta-analyses
Source: BMC Public Health. 2018 Oct 5;18:1160. doi: 10.1186/s12889-018-6062-9 (PMC6173936; doi:10.1186/s12889-018-6062-9)
Supplement: Supplementary file 5 — Table 4. Risk of bias for included studies. (DOCX 13 kb) [file 12889_2018_6062_MOESM5_ESM.docx]

**Additional file 5.** Table 4: Risk of bias for included studies.

|  | A | B | C | D | E | F | G | H | I |
| --- | --- | --- | --- | --- | --- | --- | --- | --- | --- |
| Bennett et al., 2007 | + | + | - | - | + | ? | ? | + | + |
| Conn et al., 2003 | ? | ? | - | + | + | + | + | + | + |
| Greaves et al., 2008 | + | + | - | + | + | ? | + | + | + |
| Groeneveld et al., 2011 | + | + | - | ? | + | + | + | + | + |
| Janssen et al., 2014 | + | ? | - | + | + | ? | + | ? | ? |
| Knittle et al., 2015 | + | + | - | + | ? | ? | + | + | + |
| Lakerveld et al., 2013 | + | + | - | + | - | - | + | + | + |
| Marques et al., 2017 | + | ? | - | - | + | + | + | + | + |
| Martens et al., 2012 | + | + | - | - | ? | + | + | ? | ? |
| Murphy et al., 2013 | + | ? | - | + | + | + | + | + | - |
| A: random sequence generation (selection bias). B: allocation concealment (selection bias). C: Blinding of participants (performance bias). D: Blinding of outcome assessment (detection bias). E: Incomplete outcome data (attrition bias). F: Selective reporting (reporting bias). G: Baseline similarity. H: Compliance. I: Co-interventions.  +: low risk of bias. ?: unclear risk of bias. -: high risk of bias. | | | | | | | | | |

Reference:

Balshem H, Helfand M, Schunemann HJ, Oxman AD, Kunz R, Brozek J, Vist GE, Falck-Ytter Y, Meerpohl J, Norris S, Guyatt GH: **GRADE guidelines: 3. Rating the quality of evidence.** *J Clin Epidemiol* 2011, **64:**401-406.
